# Development of a Computer Writing System Based on EOG

**DOI:** 10.3390/s17071505

**Published:** 2017-06-26

**Authors:** Alberto López, Francisco Ferrero, David Yangüela, Constantina Álvarez, Octavian Postolache

**Affiliations:** 1 Departamento de Ingeniería Eléctrica, Electrónica, Computadores y Sistemas, Universidad de Oviedo, Campus of Gijón, 33204 Gijón, Spain; UO181549@uniovi.es (A.L.); UO180830@uniovi.es (D.Y.); tina@uniovi.es (C.Á.); 2 Instituto de Telecomunicações, ISCTE-IUL, Av. Rovisco Pais, 1, 1049–001 Lisboa, Portugal; poctav@alfa.ist.utl.pt

**Keywords:** electro-oculography (EOG), eye movement, denoising, human-computer interface (HCI), signal acquisition, wavelet transform

## Abstract

The development of a novel computer writing system based on eye movements is introduced herein. A system of these characteristics requires the consideration of three subsystems: (1) A hardware device for the acquisition and transmission of the signals generated by eye movement to the computer; (2) A software application that allows, among other functions, data processing in order to minimize noise and classify signals; and (3) A graphical interface that allows the user to write text easily on the computer screen using eye movements only. This work analyzes these three subsystems and proposes innovative and low cost solutions for each one of them. This computer writing system was tested with 20 users and its efficiency was compared to a traditional virtual keyboard. The results have shown an important reduction in the time spent on writing, which can be very useful, especially for people with severe motor disorders.

## 1. Introduction

The eyes and their movements are an essential element used to express the desires, emotional states, and needs of people. For those people with major disabilities, the detection of eye movement can be an alternative form of communication.

In recent years, considerable effort has been devoted to researching methods that allow the development of robust systems for the detection and monitoring of the eyes. There are many different methods for eye tracking [1,2,3]. However, research is still ongoing to find a method that can be used in all applications of eye tracking. Once this information has been processed properly, it can be used for controlling devices such as virtual keyboards [4], powered wheelchairs [5,6,7], robots [8,9], medical applications [10,11], etc. An important field of application is the development of assistive technology oriented to various disabled people through the development of human computer interfaces [12,13,14,15,16]. These kinds of people retain the control of their eye movements, which can be translated into commands.

The main eye tracking techniques that currently exist are those based on video-oculography (VOG) and electro-oculography (EOG). In VOG-based systems, a camera is set to focus on one or both eyes and records eye movement. Although in recent years this technology has been getting cheaper, in this work, electro-oculographic technology is preferred because of its features. Firstly, EOG signals can be acquired using cheap and simple electrodes. The signals are proportional to the eye gaze displacements and easily distinguishable from other biopotentials. Besides, EOG signals are very fast, thus real time implementation is possible for translating eye movements into commands. With regards to the hardware requirements, devices based on EOG signal acquisition are as cheap as those based on the VOG method. Moreover, this technique has a wide ranging eye tracking capability: the field-of-view is not restricted to a video camera or sensors [2]. Because of these characteristics, EOG signals properly recorded by a wearable device are appropriate for the development of a Human-Computer Interface (HCI).

**Figure 1:**
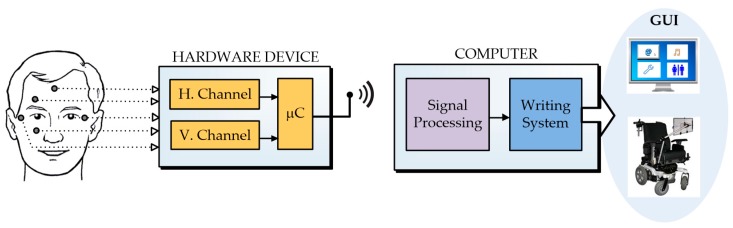
General view of the proposed system based on eye movements.

In this paper, a HCI based on ocular movement is proposed. A system of this type consists of three main elements: a hardware device for acquiring electro-oculographic signals, a software application for data processing, and a graphical user interface that allows the user to interact with the system. Figure 1 shows a simplified view of the proposed system. The hardware device acquires the electrical signals corresponding to the horizontal and vertical movement of the eyes by means of the electrodes, amplifies these signals, digitizes them, and sends them wirelessly to the computer for processing. The software application performs two main functions; one is responsible for minimizing the noise that could not be eliminated in the hardware and for this task it is very appropriate to use the wavelet transform method. The second function is to classify the EOG signals in order to clearly identify the different types of eye movements. Finally, the graphical user interface is based on a novel writing system specially designed for users with major motor disabilities, but who have intact eye movement faculties.

The rest of the paper is organized as follows. Some previous work is covered in Section 2. Section 3 provides a background to electro-oculography. Section 4 describes the hardware device. Section 5 is devoted to explaining the structure of the software application. Section 6 presents the wavelet transform for signal denoising. Section 7 presents a simple way to classify the EOG signals. In Section 8 and Section 9, the test protocol and the experimental results are presented. Finally, Section 10 draws the conclusion.

## 2. Related Work

In the literature, one can find a variety of studies with different signal acquisition devices and a few functional GUIs in the context of EOG-based HCIs. Reviewing the related literature, it can be seen that most of the research has focused either on the hardware stage or on the software stage, both entailing hard work and extensive consideration. Some of the related works reported in the last decade are presented below.

If the papers are analyzed chronologically, the oldest one [17] presents a hardware system composed of three stages: signal conditioning, pattern recognition, and command output, to process EOG signals. As an addition to the previous one, a GUI is provided in [18], but it is oriented to play a game with eye-control view. The first system similar to that proposed here is a portable wireless system (based on Zigbee), which incorporates a virtual keyboard and a patient assistant. The keyboard only incorporates the alphabet and numbers by assigning a key for each of them [16]. In the same way, other researchers have developed a GUI that includes a virtual keyboard to write a message and a module to request personal needs [4]. The GUI incorporates some special characters, but their design is like that of a traditional keyboard. The signal processing stage and dimensions of the non-portable hardware device are the main weaknesses of this work. As an improvement on previous research, the GUI is projected onto special eyeglasses, which at the same time, hold the electrodes for signal capturing [14]. The interface allows eight different commands to be selected for patient assistance, but does not use a keyboard as a communicative tool. The continuous wavelet transform (CWT) and a neural network are employed for signal processing. The design and application of a user interface was also discussed in [19], where a multimodal interface that combined mental activity and eye movement to displace a dot in a simple GUI was developed. This interface allows the study of an increase in the number of commands that a user can generate in order to interact with a device.

Most recently, the prototype called PANTOJO focused only on the hardware stage and presented a compact gaze tracker in which the electrodes are secured in an elastic head band [20]. Another interesting piece of research is the comparison between the techniques proposed by R. Barea and A. Bullings’ works [21]. Additionally, the authors suggested several potential improvements in those works and investigated three different target configurations on the screen. In the literature, research related to novel signal classification methods can also be found [22]. Related to the development of writing systems, a system with a virtual keyboard can be found on which the letters and numbers are separated into nine groups [23]. This offers an improvement over a system that only allows writing one letter, in which a key is dedicated to each one of them [24]. The writing speed obtained is almost double.

In recent years, more research in this field has become available. However, the papers are mainly focused on acquiring EOG signals with a greater quality. For example, eye gestures were analyzed again for the interface and control of appliances [25] and the solution to the drift problem [26] was also revised. Other research can be found where a novel encoding paradigm to convey users’ intentions is presented as a preliminary step to develop an HCI [27]. The only EOG-based GUIs oriented to physically disabled persons that can be found in the last three years are [28] and [29]. The first one only uses horizontal eye movements and is designed using MATLAB. The GUI is a small liquid crystal display (LCD) that contains some buttons to help a user to express what he/she wants through messages. The second system presents a better GUI in which a user can type text using a virtual keyboard. Its acquisition system is based on Arduino interfaced ADS1299 with a wearable dry electrode mask used to record and process EOG signals. All the eye movements and blinks are classified by the voltage thresholds.

As can be seen, the development of EOG-based HCIs has received some attention in recent years. However, there are few EOG-based devices available for patients which are both simple to use and affordable. They are all restricted to academic research and are not commercially available. The only commercial devices that can be found today are Cyberlink [30], Biomuse [31], the Bionomadix EOG Amplifier [32], and the BlueGain EOG Biosignal Amplifier [33], whose only purpose is signal acquisition and wireless transmission. These systems could be combined with external hardware to develop an HCI; however, these devices have not reached the mass market yet. In addition, some of these existing systems are limited by their wires and bulky designs [22].

The novelty of this work is the writing system developed based on crossheads instead a traditional keyboard layout. These two types of systems have been compared in terms of writing speed, precision, and the information transfer rate to highlight the advantage of the system presented here. In addition, the main elements surrounding these kinds of systems have been analyzed and solutions have been proposed for each one.

## 3. Electro-Oculography

Electro-oculography is a technique that enables the detection of eye movement. This is because the eye can be modeled as a dipole, with the cornea and the retina being the positive and the negative potentials, respectively. With the movement of the eyeball, an electric field is generated, which can then be measured. The EOG amplitude varies from 50 to 3500 µV and its frequency ranges from DC to 50 Hz [34,35]. However, an EOG signal is not deterministic. It depends on many factors: the placement of electrodes, skin-electrode contacts, environmental conditions, other biopotentials, etc. To obtain robust results, adequate acquisition and processing modules must be developed.

**Figure 2:**
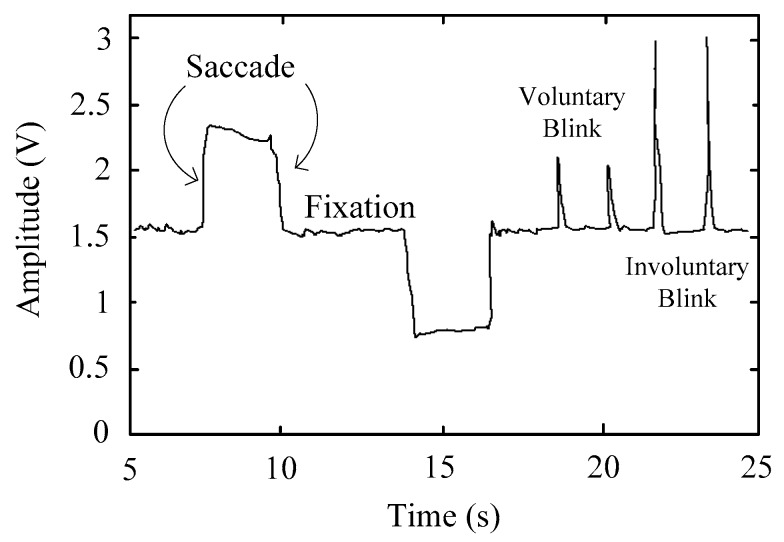
EOG signal showing the basic eye movement types.

Three types of eye movements can be recognized in the EOG signal: saccades, fixations, and blinks (Figure 2). The first one is the most important for this work because it represents the simultaneous movement of both eyes. They are characterized by a maximum velocity of 400 °/s, and an amplitude of 20°. The amplitude of saccadic movements is practically linear for gaze angles of ±30°, with a sensitivity range from 10 to 30 µV for each degree of eye movement [36]. Fixations are the state during which the gaze is focused on a particular location. Blinks are classified into two categories: involuntary and voluntary. The latter is related to monocular blinks and has an amplitude that is 10 to 20 times greater than involuntary blinks that are related to ordinary mutual blinking [37]. Eye blinks could play a key role in the control of the system if they are performed as a validation signal [38,39,40].

## 4. Hardware Device

**Figure 3:**
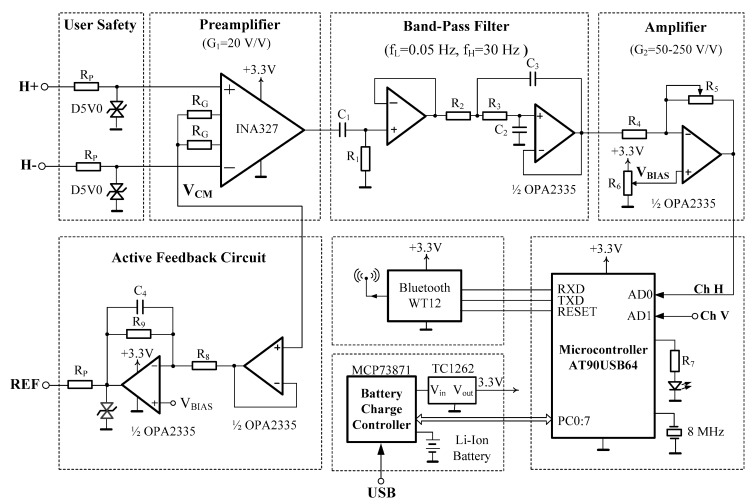
Electrical schematic of the hardware device (only the horizontal channel is shown).

For the acquisition of electro-optical signals, it is necessary to have a hardware device to capture the signals and send them to the computer. Due to the high cost of existing commercial equipment, it was decided that a low cost hardware device should be developed. This section describes the most noteworthy aspects of the electronic design. Other alternatives can be consulted in the literature [4,14,16,41]. Regarding medical instrumentation, there are two excellent books for the design of acquisition systems [42,43]. Figure 3 shows the electrical schematic of the device.

This device is responsible for capturing the EOG signals corresponding to the horizontal and vertical movement of the eyes, amplifying, filtering, and digitizing them to send them wirelessly to the signal processing module located on the computer.

The EOG signals are acquired by surface electrodes located at specific positions on the face. These electrodes convert the ion currents into a stream of electrons. Ag/Ag-Cl electrodes are typically used. These types of electrodes are the same as the ones used for the acquisition of other types of biopotentials (ECG, EEG, EMG). The number and location of these electrodes are not unique and affect the properties of EOG signals [44,45]. In this work, five electrodes are used: two electrodes to acquire horizontal eye movements (H+, H−), two for vertical movements (V+, V−), and a fifth electrode that will serve as a reference electrode (REF). The security of the user and the device is the most important issue. The designer must consider all scenarios and meet regulations. The most important standard to take into account is IEC-60601 [46], which limits the current though the electrodes to less than 10 µA rms. To ensure this limit, a 330 kΩ resistance is placed in the signal path. It is a widely used low cost solution. One of the most dangerous scenarios is the overvoltage from a defibrillator. To protect the user against this kind of overvoltage, a transient suppressor diode (TVS) is placed between each electrode and the ground. These diodes operate by suppressing all of the overvoltage above their breakdown voltage and by shunting excess current. A more expensive solution to achieve galvanic isolation is to use an isolate amplifier between the instrumental amplifier (IA) and the filter stage. A typical solution is to use the CI ISO124. On the other hand, it is recommended that the process of charging the battery is done without the battery in use.

The electrodes are characterized by low values of electric potential and high output impedance. Therefore, the amplification step has to have a very high input impedance and a high common mode rejection ratio (CMRR). Instrumentation amplifiers (IA) fulfill these two premises, so they are the components most used to amplify these types of signals.

The proposed EOG-based computer writing system is oriented to physically disabled people. This type of user has a very limited mobility because they are people who have damaged the motor system. This behavior also conditions the design of the EOG filtering stage, which consists of DC drift elimination and interference suppression. In order to obtain an appreciable attenuation, high order filters are desirable. In this work, the aim for this stage is to achieve a trade-off between attenuation and hardware simplicity. Specifically, after the instrumentation amplifier stage, a second order band-pass filter could be adequate. At this point, a high-pass filter (HPF) with a cutoff frequency of 0.05 Hz is added to remove the dc component. Next, a second order or higher active low-pass filter (LPF) with a corner frequency of 30 Hz is added to avoid aliasing out-of-band noise and also to minimize the 50/60 Hz power line interference.

The signal without a DC component is regained to achieve the total gain. The full-scale voltage of the ADC is 3.3 V, which implies a total gain of approximately 3300 V/V (assuming a 1 mV input signal). This total gain is distributed between the IA and an additional gain amplifier. The operational amplifier used for this stage must be very low noise and low power (OPA2335), so that it does not dominate the noise of the system and can be used for battery-power systems.

The last gain stage is followed by a multiplexer block (MUX) that feeds into a 10-bit ADC. The MUX and the ADC are embedded in an 8-bit microcontroller device (AT90USB1287). The sampling frequency of the ADC is one of the key factors in the performance of the proposed system. The higher the sampling rate, the greater the leeway when it comes to treating the obtained signal digitally, and therefore, the system’s precision will be greater and its writing speed will be lower.

The frequency range of the EOG signal is typically between 0 to 50 Hz. According to the Nyquist criterion, the sampling frequency must be at least twice the maximum frequency component in the signal to avoid the aliasing phenomenon. Therefore, a sampling frequency of at least 100 samples per second is needed. The ADC supports a sampling rate of up to 15.38 kHz.

The ADC output is carried to the Bluetooth device (BlueGiga WT12) through the microcontroller USART (Universal Synchronous and Asynchronous serial Receiver and Transmitter). In this module, the EOG signals are coded according to a format of 10 characters of test: XXXXYYYY\r\n, where Xs and Ys represent a decimal value of the horizontal and vertical channel, respectively, “\r” is the carriage return, and “\n” is the line break. Each character is coded by 8 bits, according to the UTF-8 standard. For instance, XXXX can be between 0000, when there is no eye movement, to 1023, when the eye movement is maximum. This range is because the ADC is of a 10-bit resolution, so its output can achieve 1024 different outputs codes, from 0000 to 1023.

The transmission rate (bauds rate) of the USART module can be obtained from the sample rate of the EOG signal: 100 samples/sec × 10 characters/sample × 8 bits/character = 8000 bps. The USART bauds rate closer to 8000 bps is 9600 bps. For this value, the corresponding sample rate is: 9600 bits/s × 1 character/8 bits = 1200 characters/s = 1200 bauds and 1200 characters/s × 1 sample/10 characters = 120 samples/s. This is the sample rate at which each channel is scanned by the ADC.

Regarding the communication between the Bluetooth and the computer, no additional requirements are needed because both devices support speeds of up to 115200 bps.

A 3.3 V power supply is obtained from the USB connector by means of a low dropout regulator (Microchip TC1262). The circuits are supplied by a 3.7 V/2000 mAh Li-ion battery. Battery charge management is achieved via a Microchip MCP73871 controller. An estimation of the battery life can be ascertained by dividing the battery capacity (mAh) by the average current consumed by the device. The average current depends on the number of different device states, the average current in each state, and the time spent in each state. For instance, the microcontroller has five states: active, power-down, power-save, Idle, and standby, and it is very difficult to know the time spent in each state. It is possible to achieve realistic results considering the current in the active state and multiplying the battery life by 0.7. In our case, the battery capacity is 2200 mAh, and the average current of all devices in an active state is around 205 mA (I_µC_ = 70 mA, I_Bluetooth_ = 60 mA, I_IA_ = 25 mA, I_OA_ = 50 mA). Thus, the estimated battery life is about 7.5 h. To reduce the power consumption of the Bluetooth device, it would be better to use Bluetooth Low Energy (BT4.0 LE), whose power consumption is around 20 mA.

**Figure 4:**
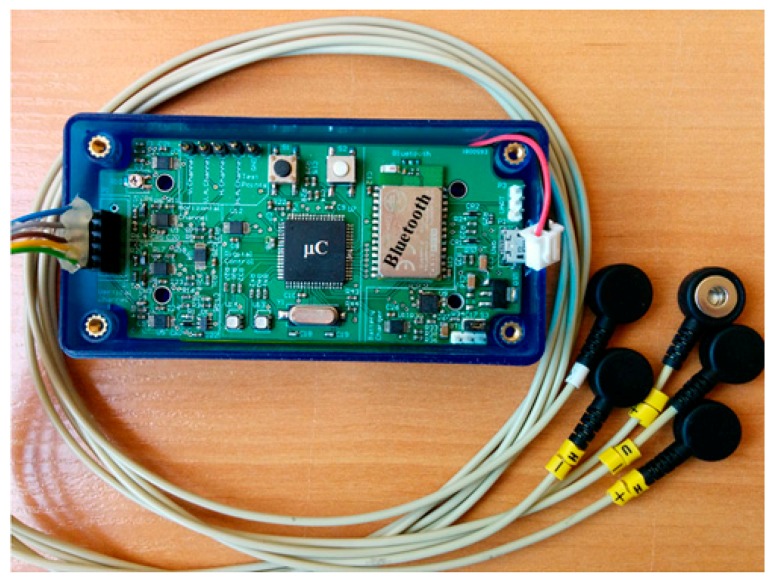
Photo of the hardware prototype (the battery is under the printed circuit board).

The device is implemented on a 10 × 5 cm printed circuit board. Figure 4 shows the device with the leads. As can be seen, the signals still require more processing to eliminate the noise present in them.

## 5. Software Application

**Figure 5:**
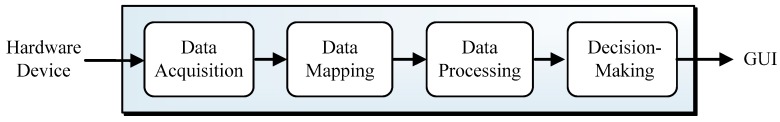
Block diagram of the software application.

Figure 5 shows the block diagram of the developed software application. It consists of four modules: data reading, data mapping, data processing, and decision making. The data acquisition module reads data (bytes) from the Bluetooth device and sends them to the mapping module. The received data has to be divided into EOG samples (each one occupies a line of text of the communication) and in each EOG sample, the two channels, horizontal and vertical, must recognize each other.

In the data processing module, samples received from the mapping module (already formatted in strings with the channels separated) are processed to determine which movement has been executed with the eyes, from the range of five movements available (those for which the application was designed); gaze up or down, left or right, or blink. Within the data processing module, the tasks dedicated to noise elimination and signal classification are especially important, so these tasks will be the subject of a special study in Section 6 and Section 7, respectively. Finally, the decision module receives the data from the processing module and prepares the movement of the cursor or the execution of a click.

The graphical user interface (GUI) allows the user to write on the computer by exclusively using the movements of the eyes. The GUI was designed using the QtCreator “design” mode, which enables widgets to be added manually. This mode facilitates the graphical presentation and the architecture of slots which is executed on emitting the signal that indicates that the buttons have been pressed [47].

**Figure 6:**
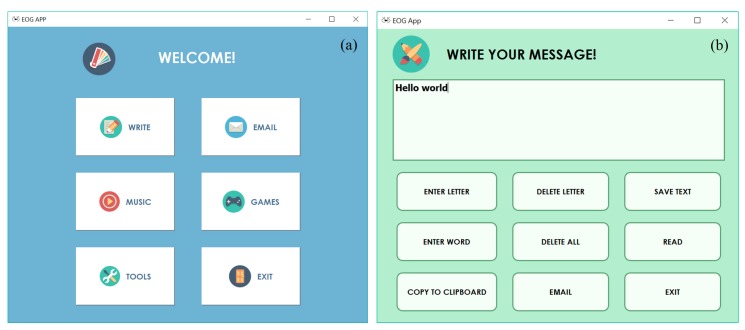
(**a**) Main menu of the application; (**b**) Writing module.

Figure 6a shows the main menu of the application. It consists of six modules: writing, sending email, music player, games, setting, and exit. Figure 6b shows the writing module developed. It consists of nine buttons with different functionalities (enter letter, delete letter, save text, etc.), along with a text edit box where the text entered by the user will appear. The user controls the cursor through eye movements, until arriving at the button corresponding to the function to be executed which is selected by a blink of the eye.

**Figure 7:**
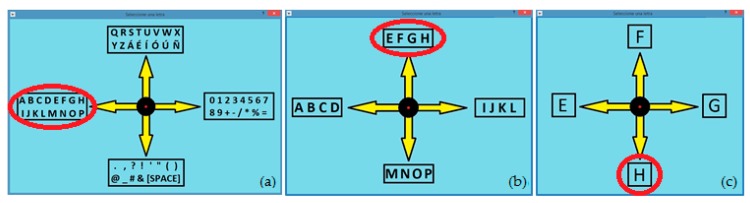
Selecting a character using three basic eye movements.

To start typing, the user has to select the “ENTER LETTER” button and then a crosshead system (Figure 7a) will appear. To select any alphanumeric character of the 64 available, the user only has to perform three basic movements of the eyes. For example, to enter the letter “H”, the user must first look to the left, which will display the group of letters in Figure 7b. Then, the user should look upwards and the group of letters in Figure 7c will be displayed. Finally, looking down, the user will select the letter “H”. Immediately it will appear in the text box and the crosshead system will return to the first stage to continue writing. Once the “ENTER LETTER” button is selected, the writing process is maintained, being compatible with the selection of other functionalities of the writing module. It is necessary to select the button again so that the crosshead system disappears.

In order to enable communication not only by writing, but also by speech, a button has been added that enables, by means of a voice synthesizer, the text entered to be read aloud. For this purpose, the Microsoft Speech APIs (SAPI 5.4) from Microsoft [48] have been used, which enables the reading of text by means of Text To Speech (TTS) technology, using the voice libraries provided by Microsoft.

## 6. Signal Denoising

Most interference was removed in the hardware device; however, wideband noises and the baseline drift are not easily suppressed in the hardware stage. It is more effective to remove them through the use of software. The EOG signal is non-stationary (its spectrum varies over time) in such a way that many of its temporal aspects, such as the beginning and the end or the instant of appearance of a singularity, cannot be adequately analyzed with the Fourier transform, or with the Fourier transform with window (FTW). For these types of signals, the wavelet transform (WT) is able to concentrate better on transient and high frequency phenomena. This mathematical tool has been used in other works with ECG signals [49,50] and EOG [14,51,52]. In this work, a systematic study to reduce noise in EOG signals by using WT is presented. First, a brief introduction to WT is necessary.

The WT of a function f(t) is the decomposition of f(t) into a set of functions $\psi_{s\tau }\left(t\right)$, which form a base and are called the “wavelets” [53]. The WT is defined as:

(1) $$WT_{s,\tau }=\int_{-\infty }^{\infty }f\left(t\right)\psi_{s,\tau }^{*}\left(t\right)\;dt$$

The wavelets are generated from the translation and scaling of the same wavelet function ψ (t), called the “mother wavelet”, is defined as:

(2) $$\;\psi_{s,\tau }\left(t\right)=\frac{1}{\sqrt{\left|s\right|}}\psi \left(\frac{t-\tau }{s}\right);\;s,\tau \;\in \;\mathbb{R};\;s\;\neq 0$$

where s is the scale factor and τ is the translation factor.

**Figure 8:**
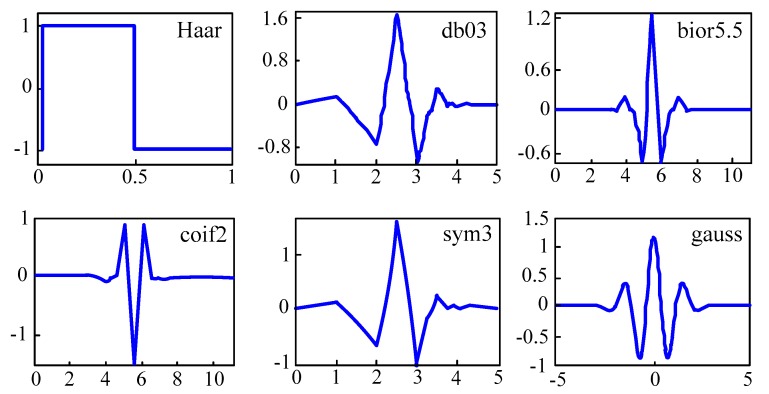
Examples of wavelet families.

The wavelets $\psi_{s\tau }\left(t\right)$ generated from the same mother wavelet function ψ(t) have a different scale s and location τ, but all have the same shape. Figure 8 shows several families of wavelets that have proven to be especially useful for EOG denoising. The abscissa axis represents the time in seconds, while the ordinates one is the normalized amplitude. The index number refers to the number of coefficients. Each wavelet has a number of zero moments or vanishing moments equal to half the number of coefficients. For example, the Haar wavelet has one vanishing moment. A vanishing moment limits the wavelets’ ability to represent polynomial behavior or information in a signal.

Changing the value of s covers different frequency ranges. The function f(t) can be reconstructed from the discrete wavelet coefficients $WT_{s,\tau }$:

(3) $$f\left(t\right)=A\underset{s}{\sum }\underset{\tau }{\sum }WT_{s,\tau }\psi_{s,\tau }\left(t\right)$$

where A is a constant that does not depend on f(t). These continuous wavelet functions with a discrete scale and translational factors are called discrete wavelets (DW). The analysis is more efficient by choosing scales and translations based on powers of two.

**Figure 9:**
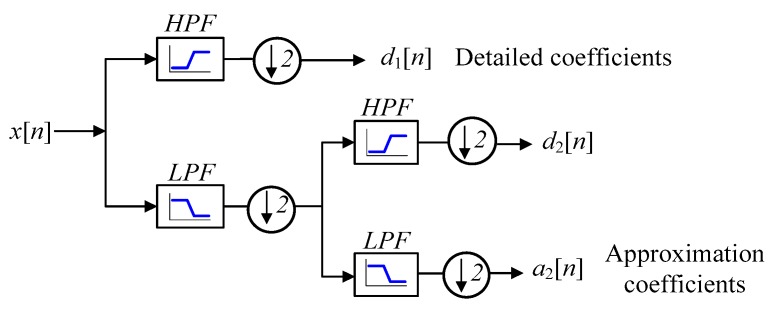
Wavelet decomposition tree using two levels of decomposition.

In order to obtain the DWT of a digitalized signal x[n], iterative signal decomposition consisting of low-pass and high-pass filters is used. Figure 9 shows the wavelet decomposition tree using two levels of decomposition. The detail coefficients $d_{j}$ have low values and contain high-frequency noise. On the other hand, the approximation coefficients $a_{j}$ have high values and contain much less noise than the original signal [54].

The elimination of noise using the WT method consists of three stages: (1) Wavelet decomposition of an input noisy signal; (2) Threshold estimation and thresholding of wavelet coefficients; and (3) Inverse wavelet transform for the reconstruction of a denoised signal. The decomposition step consists of applying the WT to noise-contaminated signals to obtain WT coefficients. The selection of a particular wavelet defines the specific response for singularities. The wavelet coefficients resulting from the wavelet transformation correspond to a measurement of the EOG components in this time segment and frequency band. The large coefficients correspond to eye movements and the low ones to the noise. The decimation stage that is found after the filter makes it time variant. The non-decimated WT (UWT) does not perform this decimation process, but rather modifies the filters by interpolating zeros depending on the decomposition level. The filters at each level perform the inverse decimation (up-sampling) process by which zeros are inserted between every two samples. In this way, the UWT has the same number of coefficients at each level, which is equal to the number of coefficients of the signal being analyzed [55,56,57,58].

Thresholding consists of selecting an adequate threshold value for the WT coefficients to remove those with small values. Thresholding methods can be classified into hard and soft, to remove or attenuate the spectral components of lower weight. The most important threshold rules for wavelet coefficients are Minimax, Universal, and Sure (Heuristic and Rigorous) [59]. The first two methods only depend on the estimated noise level and the length of the data sequence, whereas the latter is an adaptive threshold, which is calculated for each particular data sequence. These thresholds have been extensively studied and provide near optimum results in many situations. For these reasons and for the ease of application, they are used in this work.

In the last step, the original signal is reconstructed using the inverse wavelet transform. The process is reversed. The WT coefficients are convolved both with the reconstruction scaling and the reconstruction wavelet filters [60]. Then, these results are added together to obtain the denoised EOG signal. The thresholding of wavelet coefficients greatly affects the quality of EOG morphology; thus, threshold determination is an essential issue in this case.

**Figure 10:**
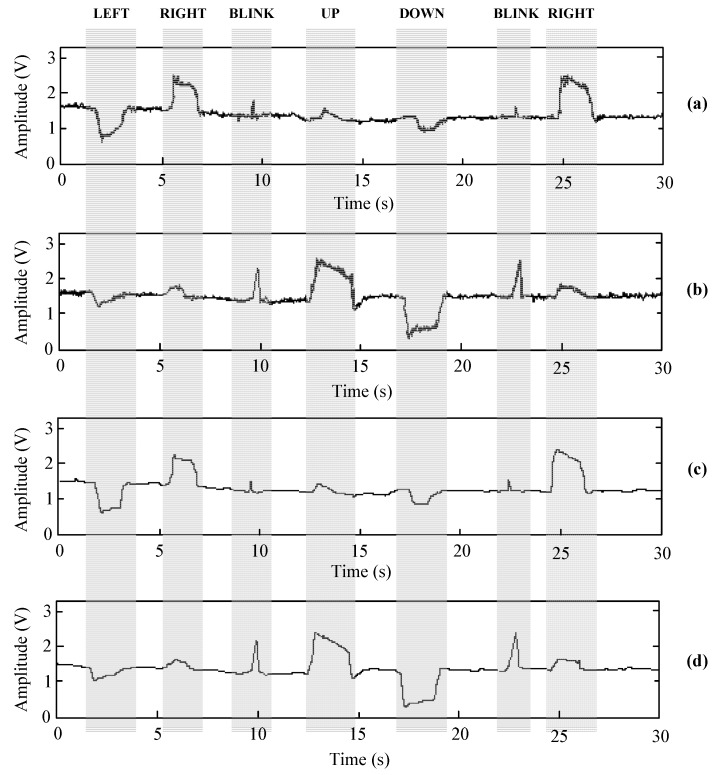
Waveforms of the raw and filtered EOG signals using UWT bior5.5 wavelet and Minimax thresholding: (**a**) Raw EOG signal obtained from horizontal eye movement. (**b**) Raw EOG signal obtained from vertical eye movement. (**c**) Denoising of raw EOG signal obtained from horizontal eye movement. (**d**) Denoising of raw EOG signal obtained from vertical eye movement.

To assess the effect of noise on a signal, two parameters are commonly used: the signal-to-noise ratio (SNR) and the mean square error (MSE). These parameters are defined as follows:

(4) $$SNR_{dB}=10\log\left[\frac{\sum_{n=1}^{N}{\left(x\left[n\right]\right)}^{2}}{\sum_{n=1}^{N}{\left(x\left[n\right]-x_{R}\left[n\right]\right)}^{2}}\right]$$

(5) $$MSE=\frac{1}{N}\overset{N}{\underset{n=1}{\sum }}{\left(x\left[n\right]-x_{R}\left[n\right]\right)}^{2}$$

where n is the length of the EOG signal, x[n] is the original signal, and $x_{R}\left[n\right]$ is the reconstructed signal. Tests have proved that by using UWT with the bior5.5 wavelet and the Minimax threshold, an SNR of almost 20 dB is obtained. The analysis was performed using the LabVIEW Advanced Signal Processing Toolkit. Figure 10 shows the waveforms of the raw and filtered EOG signals using UWT bior5.5 wavelet and Minimax thresholding. These waveforms were obtained using the developed hardware prototype. Table 1 shows the value of these parameters when denoising the EOG signal using DWT and UWT with different wavelets and thresholds. As you can see in Table 1, the bior family contains the best mother wavelets due to the correlation between the shapes of the mother wavelets and EOG signals.

## 7. Signal Classification

**Figure 11:**
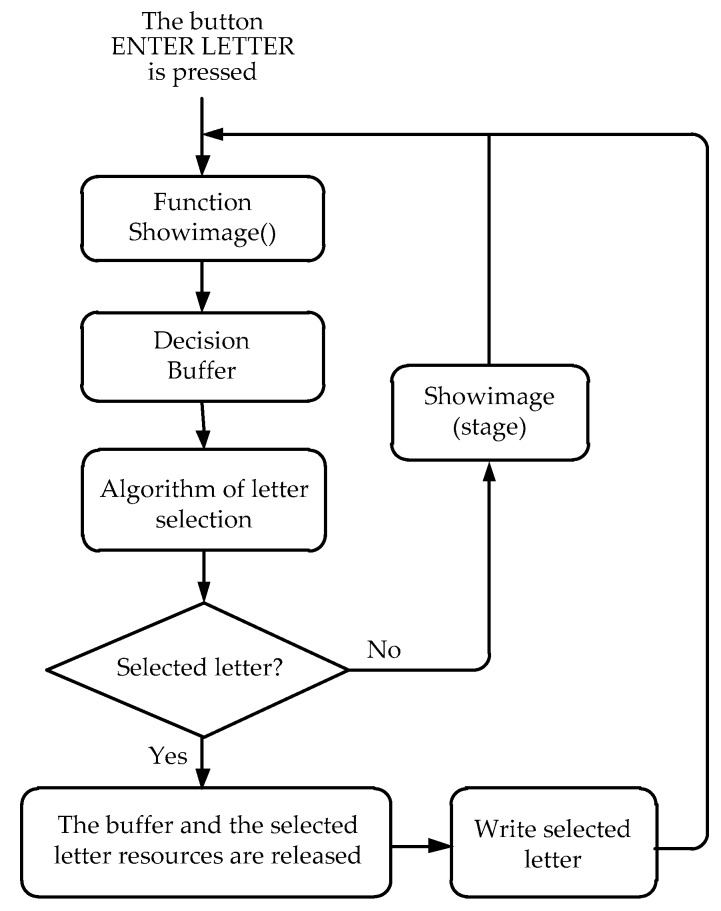
Flowchart of the writing process.

To detect the direction of eye movement, an EOG signal is classified into five patterns using a voltage threshold: left, right, up, down, and straight. These threshold values are accomplished experimentally after using a loop of adaptive thresholding, contrary to the standard fixed threshold, which is different for each user. The use of thresholds allows distinguishing between signal and noise. When the threshold values are exceeded in any of the four directions, the cursor moves in the corresponding direction. These thresholds are different for horizontal and vertical channels and each user, so they must be fitted for each situation. Thus, before using the proposed system, it is necessary to record the user’s physiological parameters. These are then used to set the threshold values. The voluntary blinking pulses are also detected using thresholding. While typical involuntary blinking is symmetric due to the simultaneous movement of the eyelids, voluntary blinking is asymmetric. The latter is easily distinguishable in the EOG signal because it shows a sharp peak with a greater amplitude than involuntary blinking and looking upwards. Figure 11 shows a simplified flowchart of the writing process to understand how the classification algorithm works.

The writing function uses several auxiliary functions that serve the main algorithm. The “Showimage()” function shows the crosshead corresponding to the iteration stage, taking into account the previous selection, if applicable. At the same time as the image is displayed, an auxiliary buffer begins storing the received samples of the digital signal processing threads. Here, it is decided in which direction the eye is being directed considering that the established thresholds are exceeded during a set time. In order to measure this time in the program, it is necessary to know the frequency at which the EOG samples are received and to make a simple calculation to determine the number of samples required. A voluntary blink, if not treated differently, will be recognized as if the user were looking upwards. However, it is known that an involuntary blink lasts between 200 and 300 ms [36]. So, the movements lasting more than 300 ms are considered as an “UP” command or as a voluntary blink if the amplitude is greater. Therefore, as in this work, the only interest is in voluntary blinks and the movements of looking upwards, and the thresholds (of the four coordinates and a blink) have been exceeded during at least 36 samples (i.e., 300 ms). So, it can be considered a cursor movement or selection motion in the crosshead system. Then, the direction of the gaze is sent to the character selection algorithm, in order to scroll through the crosshead system. The corresponding crosshead with the groups of characters that are being selected will be shown. Once a character is selected in the third stage of the crosshead system, it is displayed in the text box and returned to the beginning.

**Figure 12:**
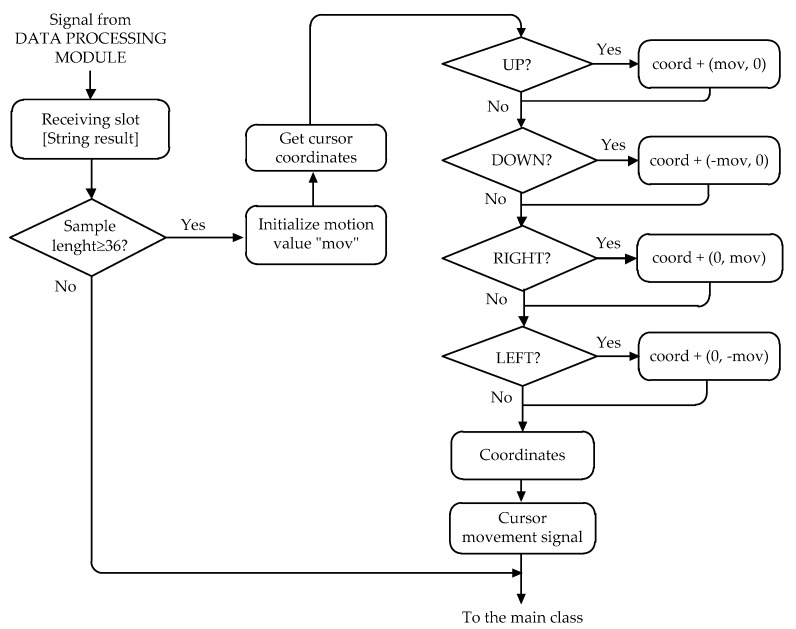
Flowchart of the algorithm that controls the movement of the cursor.

Figure 12 shows the flowchart of the algorithm that controls the movement of the cursor. The application obtains the current position of the cursor on the screen (the upper left corner being position 0,0), and depending on a predetermined jump value, the position is changed. This jump value of the cursor largely depends on the frequency at which the samples are received. A greater number of samples require a decrease of this value, so that the “sensitivity” of the mouse, or the speed, is not too much, and so that the precision is not lost. Finally, the application sends the new coordinates to the operating system, and the latter takes charge of moving the cursor to the new desired position.

This method presents a high accuracy and writing speed in sending a command to the HCI. Thus, this method is suitable for real-time applications translating potential changes due to eye movements in eight kinds of movements (up, down. right, left, up right, up left, down right, and down left) to move the cursor. The user must look at the desired key and when the cursor is positioned over the desired key, the user only needs to blink to select it. The left-click action is set by default.

The calibration of the system is necessary because EOG signals vary with each user, due to different electrode contacts, perspiration on the user’s temples, the environmental conditions, etc. [61]. This calibration also enables the reduction of long-term drifts. Thus, the HCI system requires the calibration before the beginning of the measurements. On the other hand, the user should still not be allowed to look around while using the system.

## 8. Test Protocol

**Figure 13:**
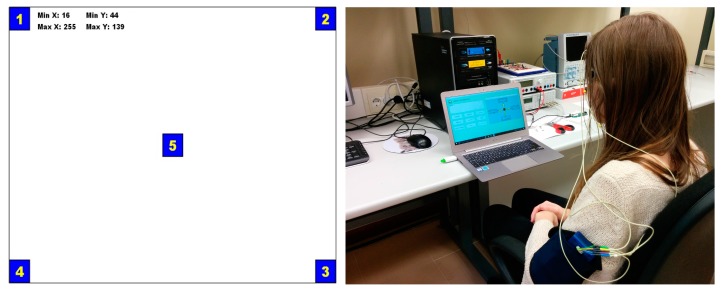
(**a**) Screen to calibrate the application; (**b**) Image of the user arrangement.

The test protocol consists of four steps:

1. **Preparation of the equipment**. This step consists of connecting all the equipment and verifying the correct operation of the system. Furthermore, all materials needed for positioning and fixing the electrodes are prepared.
2. **User preparation**. This step involves placing the surface Ag/AgCl electrodes on the user’s face. First, the face is cleaned with a gel cleaner and the electrodes are put in place. The vertical electrode is placed approximately 1 cm above the eyebrow of the right eye and 1.5 cm below the lower lid of the same eye. The electrodes of the horizontal channel are placed 1.5 cm to each side of the outer canthi. Finally, the reference electrode is placed on the user’s forehead.
3. **Calibration.** In this step, the eye movement and blink thresholds are calculated for each user. The user has to make eye movements to calculate the amplitude of his range of movements and adjust the threshold values accordingly. For this purpose, a module (Figure 13a) has been created which registers the maximum and minimum values in each axis. The user must travel with the view of the squares from 1 to 4. Once this has been done about five times, the user proceeds to look at square 5 for 5 s, recording the levels of no movement. To exit this module, the user must make a voluntary blink with the right eye, setting the selection command threshold. This procedure is always performed before using the system and lasts approximately 30 s.
4. **Experimental tests**. In this step, three experimental tests were performed. The first one tested the parameters of configuration. The next one evaluated the written mode by measuring the parameters: the speed of writing, accuracy, and information transfer ratio (ITR). Finally, the system was tested on several platforms to verify its interoperability. In the next section, these tests are described. Figure 13b shows the user arrangement with the hardware device on the left arm.

## 9. Experimental Tests

Experimental texts were evaluated by 20 volunteers: eight women and 12 men aged between 21 and 56 (mean = 27.1, sd = 9.2). They were neurologically healthy people with normal vision. In a second phase, the aim is to test the device with disabled people. None of the volunteers had any experience in operating the proposed system, nor did they have any pre-training. These experiments were admitted by the Institutional Ethics Board and all volunteers signed the consent form before starting the tests. These tests are described following:

### 9.1. Configuration Tests

To optimize the performance of the application, the following parameters were tested:

(a) **Gaze thresholds**: different thresholds of voltage have been defined in the two channels depending on the digital values received from the acquisition stage. The algorithm of the processing of the gaze determines the direction in which it has occurred.
(b) **Blinking thresholds**: Since the blink is detected in the vertical channel and results in the upward saturation of the latter, as with an upward gaze, thresholds (in time) were defined to distinguish both instructions. If the signal that generates the saturation of the vertical channel occurs in a range of 10 to 20 ms, it will be recognized as a blink and not an upward gaze.
(c) **Cursor jump**: This parameter allows you to set the speed at which the cursor moves. The coordinate update algorithm receives this parameter to update the position of the cursor in real time, so the update will occur by moving the number of positions indicated in this parameter on the corresponding axis. Five levels have been configured, setting 3 as the default level, since it is more manageable with this number of jumps per position update.
(d) **Bluetooth connection parameters**: Baud rates have been tested to adjust the number of samples per second received by the application. A rate of 1200 bauds has been set as the most optimal for handling the application.

### 9.2. Writing Tests

In [62], a low-cost system for the communication of disabled people was presented. This system uses a virtual screen keyboard that follows the format of a conventional keyboard. It covers the whole alphabet and a few special characters including “space”, as well as a return key to make corrections, if necessary. At the hardware level, this system is very similar to the system presented here. They share the analog stage. For the digital stage, the referenced work uses a commercial board (AT90USBKEY) based on the ATMEL 8-bit AT90USB1287 microcontroller. It is the same microcontroller used in the hardware device described in this work. In [62], the speed and accuracy were not tested. Both systems were compared using the same environment and the results are shown in this work.

To compare the proposed writing system (System I) with the traditional virtual keyboard (System II), the tests were conducted in the same tested environments. Five silver chloride electrodes (AgCl) were used. They were placed on either side of the eyes (H+, H−) and above and below the right eye (V+, V−) to achieve the derivation of the EOG signal of each channel. From the hardware and software point of view, a laptop with a Windows 10 Pro, Intel Core i5-4460 CPU, 3.20 GHz, 8.00 GB RAM, and 64 bits operational system was used. Twenty users were encouraged to write the same phrase, “Hello world”, 10 times.

In order to ascertain which system is better, three typical parameters were estimated: the writing speed, accuracy, and the information transfer rate (ITR). These parameters have been used by other authors in the literature and allow us to make comparisons with those devices [23,24]:

(6) $$Writing\;speed=\;\frac{Number\;of\;characters}{Process\;time}\;\left[characters/min\right]$$

(7) $$Accuracy=\;\frac{Correct\;outputs}{Total\;number\;of\;inputs}\;\times 100\;\left[\%\right]$$

To combine the writing speed, accuracy, and the number of eye movements, another parameter is frequently considered, namely the information transfer ratio (ITR) [63,64,65], defined as:

(8) $$ITR=V\left[log_{2}N+plog_{2\;}p+\left(1-p\right)log_{2}\left(\frac{1-p}{N-1}\right)\right]$$

where V is the writing speed, p is the accuracy of the subjects in making decisions, and N is the number of possible eye movements considered (four in our system: up, down, right, and left).

**Figure 14:**
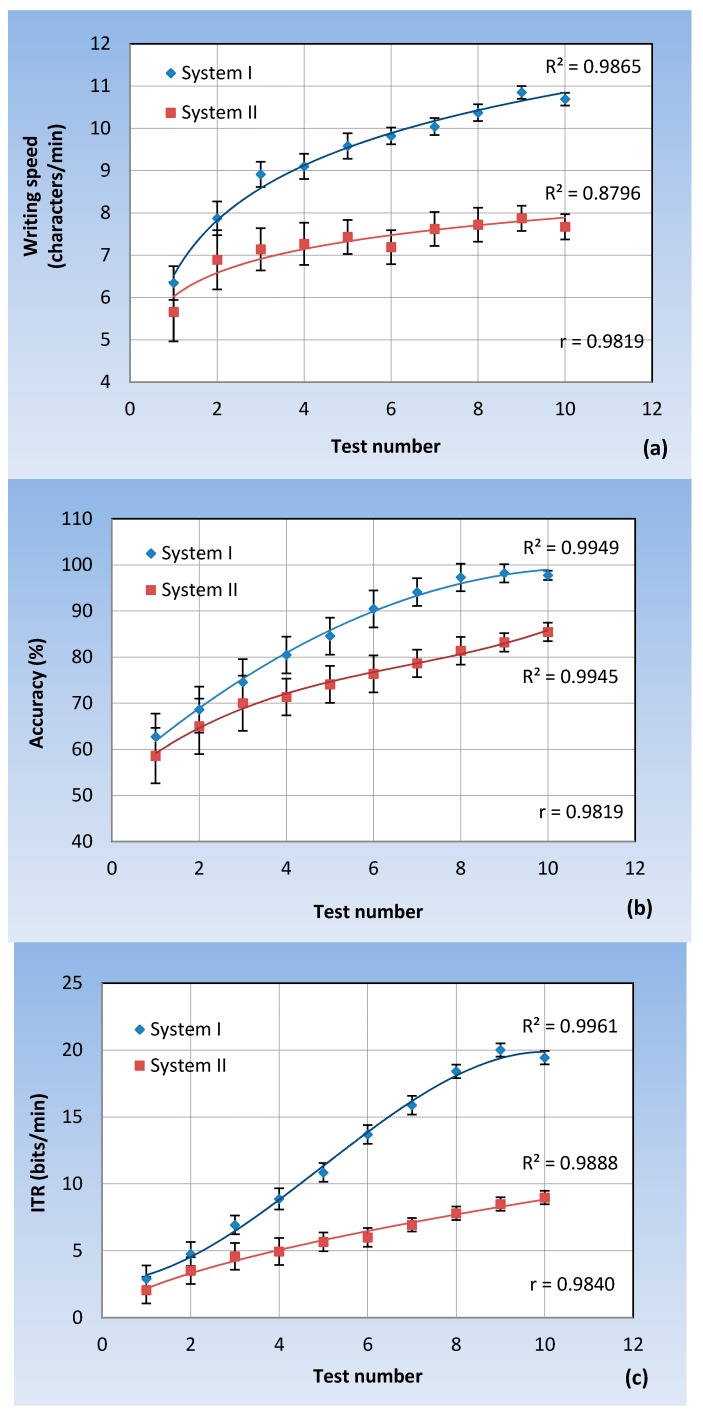
Evolution of: (**a**) the writing speed; (**b**) accuracy; (**c**) the ITR with the number of tests.

For each test, the writing speed and accuracy parameters were obtained for each one of the 20 users and then the average value was calculated. Figure 14 plots Equations (6)–(8) as a function of the number of tests. As can be seen in Figure 14a, the writing speed of System I is always greater than that of System II, with a maximum value of around 40%, and improves with the number of trials. This is a very important conclusion because System I, with adequate training, could become competitive against the video-oculography technique.

Figure 14b compares the accuracy of both systems. In the first three trials, both systems present an approximately equal accuracy. From this point on, the accuracy of System I is greater than System II, by an average value of around 10%. For the last three tests, the accuracy of System I remains steady; however, the accuracy of System II continues to increase with the number of trials, but is always below System I.

The ITR parameter evaluates the information transfer ratio (bits/min). It combines the two above parameters. As can be seen in Figure 14c, System I presents a higher transfer rate, and the more trials are done, the higher the rate.

From this study, it can be concluded that System I always presents better features than System II, even though the latter (System II) can gradually improve its accuracy and ITR with the number of trials.

Collated with the other research present in the literature, it can be seen that the system presented here offers significant improvements. The experiments carried out in [23] by writing the word “Hello” obtained an average accuracy of 95.2% and an average typing speed of only 2.37 characters/min. In the research carried out in [24], when typing five different sentences five times, an average writing speed of 5.88 characters/min was obtained. This value is almost half that obtained in our experiments. A 93% accuracy was also obtained, similar to that obtained by our system.

From the experiments carried out, it can be concluded that the classification errors mainly arise in the first tests due to a lack of practice and in the last due to fatigue. The latter is reflected in Figure 14b, in which the curve begins to slightly decrease from test nine. To obtain good results, it is important the users are focused on interacting with the system. Over time, fixation movements produce an effect called “jitter” [61]. As a consequence, users’ capacity to control their eye movements decreases inversely with tiredness. Jitter due to a loss of concentration and fatigue typically appears after 60 min of using the system.

### 9.3. Interoperability Tests

Finally, the application was tested on several operating systems: Windows 8, 64 bits (development PC); Windows 7, 32 bits (laboratory PC); and Ubuntu, 64 bits (virtual machine). The application worked as expected on all of these operating systems. The software application was also installed in three devices: a desktop computer, a laptop, and a tablet. The ability to manage the writing system is not affected by the screen size, because it is based on eye movements in the four directions in the crosshead system, not in the key selection on a virtual keyboard.

## 10. Conclusions

In this work, a computer writing system based on EOG technology is presented. A low-cost hardware device ($50) was developed to acquire and wirelessly transmit the EOG signal transmits to a computer. In addition to the electrical schematic, a discussion about the user safety, effective sampling frequency, and battery life are provided. Secondly, a software application was developed. It consists of four modules that work concurrently, facilitating real time operation. The data processing module is devoted to EOG denoise and classification. A systematic study was carried out to select the most suitable type of wavelet for EOG denoising. To detect the direction of eye movement, the EOG signal was classified into five patterns using the voltage threshold: left, right, up, down, and straight. The selection of the thresholds for each user is made by means of a previous calibration before starting to use the system. The use of thresholds allows one to distinguish between the signal and noise.

To demonstrate the efficiency of the system, it was tested with 20 users and two different virtual keyboard configurations. Regarding the writing speed, the proposed system represents an improvement of almost 40% with respect to a traditional virtual keyboard and a slightly higher accuracy, of around 10%. To obtain good results, it is important that the users are focused on interacting with the system. The capacity to control their eye movements decreases inversely with tiredness. The Jitter effect due to the loss of concentration and fatigue typically appears after 60 min of using the system.

Although this kind of system is especially designed for people suffering from severe motor disabilities, part of this work may be of interest in other applications such as wheelchair control, robot arms, or medical applications.

## Figures and Tables

**Table 1:** SNR and MSE values when denoising the EOG signal using DWT and UWT with different wavelets and threshold methods.

		DWT		UWT	
Wavelet	Threshold	SNR (dB)	MSE	SNR (dB)	MSE
Haar	SURE	11.214	0.00	11.253	0.00
	Minimax	6.388	1.27	19.015	0.15
db03	SURE	11.193	0.00	11.242	0.00
	Minimax	6.430	1.26	18.485	0.15
db08	SURE	11.177	0.00	11.242	0.00
	Minimax	16.179	1.25	19.270	0.15
db14	SURE	11.183	0.00	11.242	0.00
	Minimax	6.574	1.26	19.597	0.15
bior1.5	SURE	11.209	0.00	11.245	0.00
	Minimax	6.379	1.28	18.852	0.00
bior2.4	SURE	11.174	0.00	11.242	0.00
	Minimax	6.244	1.30	19.001	0.15
bior3.3	SURE	11.156	0.00	11.239	0.00
	Minimax	6.292	1.35	18.640	0.16
bior3.9	SURE	11.161	0.00	11.238	0.00
	Minimax	6.284	1.35	18.648	0.16
bior4.4	SURE	11.181	0.00	11.245	0.00
	Minimax	16.783	1.26	18.967	0.15
**bior5.5**	SURE	11.188	0.00	11.250	0.00
	**Minimax**	**16.659**	**1.22**	**19.664**	**0.15**
bior6.8	SURE	11.179	0.00	11.243	0.00
	Minimax	16.035	1.27	19.169	0.15
coif2	SURE	11.177	0.00	11.243	0.00
	Minimax	16.595	1.26	18.948	0.15
coif5	SURE	11.180	0.00	11.244	0.00
	Minimax	16.095	1.26	19.249	0.15
sym3	SURE	11.193	0.00	11.242	0.00
	Minimax	6.430	1.26	18.485	0.15
sym7	SURE	11.191	0.00	11.241	0.00
	Minimax	6.527	1.26	19.267	0.15
